# Next-Generation Sequencing and *In Vitro* Expression Study of *ADAMTS13* Single Nucleotide Variants in Deep Vein Thrombosis

**DOI:** 10.1371/journal.pone.0165665

**Published:** 2016-11-01

**Authors:** Maria Teresa Pagliari, Luca A. Lotta, Hugoline G. de Haan, Carla Valsecchi, Gloria Casoli, Silvia Pontiggia, Ida Martinelli, Serena M. Passamonti, Frits R. Rosendaal, Flora Peyvandi

**Affiliations:** 1 Angelo Bianchi Bonomi Hemophilia and Thrombosis Center, Fondazione IRCCS Ca' Granda - Ospedale Maggiore Policlinico and Fondazione Luigi Villa, Milan, Italy; 2 Department of Clinical Epidemiology, Leiden University Medical Center, Leiden, The Netherlands; 3 Department of Pathophysiology and Transplantation, Università degli Studi di Milano, Milan, Italy; National Cerebral and Cardiovascular Center, JAPAN

## Abstract

**Background:**

Deep vein thrombosis (DVT) genetic predisposition is partially known.

**Objectives:**

This study aimed at assessing the functional impact of nine *ADAMTS13* single nucleotide variants (SNVs) previously reported to be associated as a group with DVT in a burden test and the individual association of selected variants with DVT risk in two replication studies.

**Methods:**

Wild-type and mutant recombinant ADAMTS13 were transiently expressed in HEK293 cells. Antigen and activity of recombinant ADAMTS13 were measured by ELISA and FRETS-VWF73 assays, respectively. The replication studies were performed in an Italian case-control study (Milan study; 298/298 patients/controls) using a next-generation sequencing approach and in a Dutch case-control study (MEGA study; 4306/4887 patients/controls) by TaqMan assays.

**Results:**

*In vitro* results showed reduced ADAMTS13 activity for three SNVs (p.Val154Ile [15%; 95% confidence interval [CI] 14–16], p.Asp187His [19%; 95%[CI] 17–21], p.Arg421Cys [24%; 95%[CI] 22–26]) similar to reduced plasma ADAMTS13 levels of patients carriers for these SNVs. Therefore these three SNVs were interrogated for risk association. The first replication study identified 3 heterozygous carriers (2 cases, 1 control) of p.Arg421Cys (odds ratio [OR] 2, 95%[CI] 0.18–22.25). The second replication study identified 2 heterozygous carriers (1 case, 1 control) of p.Asp187His ([OR] 1.14, 95%[CI] 0.07–18.15) and 10 heterozygous carriers (4 cases, 6 controls) of p.Arg421Cys ([OR] 0.76, 95%[CI] 0.21–2.68).

**Conclusions:**

Three SNVs (p.Val154Ile, p.Asp187His and p.Arg421Cys) showed reduced *ex vivo* and *in vitro* ADAMTS13 levels. However, the low frequency of these variants makes it difficult to confirm their association with DVT.

## Introduction

Deep vein thrombosis (DVT) is a common, life-threatening thrombotic disease caused by both environmental [1] and genetic risk factors [2–6].

Previously, we used a tailored next-generation sequencing (NGS; SOLiD4 platform, Applied Biosystem, Foster, USA) approach [7] to sequence the coding region of 186 haemostatic and pro-inflammatory genes to evaluate the contribution of rare and low-frequency coding variants to DVT risk, in 94 Italian patients affected with idiopathic DVT and 98 frequency-matched controls [8]. Rare and low-frequency coding variants of *ADAMTS13* (minor allele frequency [MAF] <1% and <5%, respectively) were associated with DVT in a burden test. Patients carrying these single nucleotide variants (SNVs) had lower plasma ADAMTS13 activity than non-carriers. We reported an excess of rare coding variants of *ADAMTS13* in patients versus controls (16/94 vs 4/98, odds ratio [OR] 4.8), as well as for low-frequency variants (34/94 vs 23/98, [OR] 1.9) [8].

An association between rare *ADAMTS13* variants and DVT risk is plausible, due to the role of ADAMTS13 (a disintegrin metalloprotease) in the cleavage of von Willebrand factor (VWF) [9, 10]. Indeed, a mild reduction of ADAMTS13 activity may lead to a reduced cleavage activity an a consequent presence of increased levels of circulating high molecular weight multimers of VWF. Moreover, its deficiency, either genetic or acquired, leads to a severe thrombotic microangiopathy known as thrombotic thrombocytopenic purpura (TTP) [11].

A role for ADAMTS13 in the pathogenesis of arterial thrombosis has been suggested. Several *in vivo* studies have demonstrated that a reduction of ADAMTS13 activity can affect the development of arterial thrombosis [12–13].

A recent meta-analysis associated reduced ADAMTS13 levels with an increased risk of myocardial infarction [14]. Furthermore, a moderately reduction of ADAMTS13 activity was also associated to the risk of ischemic stroke [15].

In this study, we investigated the functional role of nine rare *ADAMTS13* variants (p.Val154Ile, p.Asp187His, p.Thr339Arg, p.Arg421Cys, p.Tyr603Cys, p.Asp836Gly, p.Arg925Gly, p.His1196Gln and p.Thr1249Pro) identified in eight Italian DVT patients and previously reported to be associated with DVT on burden testing [8]. Two replication studies were performed to validate the association between the three selected rare *ADAMTS13* variants (p.Val154Ile, p.Asp187His and p.Arg421Cys), which cause an *ex vivo* and *in vitro* reduction of ADAMTS13 activity and DVT.

## Materials and Methods

### Rare *ADAMTS13* SNVs Previously Identified in DVT Patients

In our previously study, we reported the association between 11 *ADAMTS13* SNVs (p.Val154Ile, p.Asp187His, p.Ala325Val, p.Thr339Arg, p.Arg421Cys, p.Tyr603Cys, p.Trp746Cys, p.Asp836Gly, p.Arg925Gly, p.His1196Gln and p.Thr1249Pro), identified in Italian DVT patients [8] and DVT. A further validation of these SNVs by Sanger sequencing was performed. Two SNVs (p.Ala325Val and p.Trp746Cys) have not been confirmed and therefore excluded from this study. To evaluate the effect of the remaining 9 SNVs on plasma ADAMTS13 levels, a further biochemical characterization of the eight patients heterozygous carriers for one of the previously reported *ADAMTS13* SNVs (patient n. 1 carried both variants p.Val154Ile and p.Thr339Arg) was performed. ADAMTS13 activity and antigen levels were measured in patients’ plasma samples using FRETS-VWF73 [16] and ELISA assays [17], respectively. FVIII coagulant activity (FVIII:C) and VWF antigen (VWF:Ag) levels were performed by previously described methods [18]. The presence of neutralizing (inhibitors) anti-ADAMTS13 autoantibodies was assessed using a FRETS-VWF73-based mixing assay as previously reported [19]. Non-neutralizing anti-ADAMTS13 autoantibodies were evaluated using the Technozym ADAMTS13 inhibitor (Technoclone, GmbH, Vienna, AU).

### *In Silico* Evaluation of ADAMTS13 Variants

To investigate the potential functional effect of each SNV, the Combined Annotation Dependent Depletion (CADD; http://cadd.gs.washington.edu/info; accessed September 2016) was used. This tool integrates different functional aspects and different annotations into a single outcome, the C-score [20]. A scaled C-score greater or equal to 10 indicates that SNV was predicted to be the 10% most deleterious substitutions, a score greater or equal 20 indicates the 1% most deleterious.

### Plasmid Construction

The ADAMTS13 wild-type (WT) cDNA was subcloned into the mammalian expression vector pcDNA^™^3.1/V5-His TOPO^®^TA (Invitrogen, Carlsbad, CA, USA). The nine *ADAMTS13* variants (p.Val154Ile, p.Asp187His, p.Thr339Arg, p.Arg421Cys, p.Tyr603Cys, p.Asp836Gly, p.Arg925Gly, p.His1196Gln and p.Thr1249Pro) were separately introduced into the pcDNA3.1-ADAMTS13 WT expression vector by site-directed mutagenesis using PFU Turbo DNA polymerase (Stratagene, La Jolla, CA, USA) and specifically designed primers. The presence of each variant was confirmed by Sanger sequencing analysis.

### *In Vitro* Expression Studies

For *in vitro* expression studies, pcDNA3.1-ADAMTS13 WT and mutant expression vectors were transiently transfected into human embryonic kidney (HEK293 cells; a gift from professor Eikenboom, Department of Thrombosis and Haemostasis, Einthoven Laboratory for Experimental Vascular Medicine, Leiden University Medical Center, Leiden, the Netherlands). HEK293 were grown in Dulbecco’s Modified Eagle’s medium (DMEM):F12 supplemented as previously reported [21]. Cells from 100-mm plates were transfected with 8 μg of plasmid DNA, in Opti-MEM Reduced Serum Medium (Invitrogen, Carlsbad, CA, USA), supplemented with 1% of L-glutamine. Transfections were performed using jetPEI (PolyPlus-transfection; Celbio, Pero, Italy), according to the manufacturer’s instructions. After 72 hours of transfection, conditioned media and cell lysates were collected. Antigen and activity levels of WT and mutant recombinant (r) ADAMTS13 proteins were analyzed in the conditioned medium in at least 3 separate experiments using ELISA [17] and FRETS-VWF73 assays [16], respectively. The amount and the activity of the mutant rADAMTS13 proteins are reported as a percentage of WT set as 100% and 95% confidence intervals (CI). The antigen and activity levels of untransfected cells were measured as negative control. The flow chart of the SNVs *in vitro* characterization is provided in S1 Fig.

### Western Blotting Analysis

The western blotting analysis was performed for those variants which showed reduced ADAMTS13 levels in both *in vitro* expression studies and patients’ plasma, except for p.Val154Ile (unavailable patient’ plasma sample) and p.Tyr603Cys (inconsistency between *ex vivo* and *in vitro* ADAMTS13 levels). WT and mutant (p.Val154Ile, p.Asp187His, p.Arg421Cys and p.Tyr603Cys) rADAMTS13 proteins in conditioned media and cell lysates were analyzed using an anti-V5 monoclonal antibody against the C-terminal tag of rADAMTS13 (Invitrogen, Carlsbad, CA, USA) followed by a peroxidase-labeled anti-mouse immunoglobulin G (Amersham Biosciences, Uppsala, Sweden) [22]. Cellular alpha-tubulin was used as control to verify equal protein loading across samples and detected using anti-alpha-tubulin monoclonal antibody (Abcam; Cambridge, MA). The amount of each mutant rADAMTS13 contained in cell lysates was normalized using the respective band of alpha-tubulin, quantified by densitometry analysis using NIH Image J software (NIH, Bethesda, MA, USA) and compared to the WT set as 100%.

### Replication Studies

Our previous study [8] was limited by the size of the population enrolled. Therefore, two replication studies were carried out to validate the association between selected *ADAMTS13* SNVs and the DVT risk. The *ADAMTS13* SNVs which showed reduced *in vitro* activity levels comparable to patients’ plasma levels (p.Asp187His, p.Arg421Cys) and p.Val154Ile (patient plasma sample was missing) were selected. The other SNVs were excluded because: (i) there was an inconsistency between *ex vivo* and *in vitro* values (p.Tyr603Cys and p.Thr1249Pro) or (ii) they showed normal *ex vivo* and *in vitro* ADAMTS13 activity (p.Thr339Arg, p.Asp836Gly, p.Arg925Gly and p.His1196Gln). For the first replication study, performed in the Italian population, the allele frequency of SNVs p.Val154Ile, p.Asp187His and p.Arg421Cys was assessed in 298 patients and 298 controls from the DVT Milan study Clinical characteristics and enrolment criteria were previously described [8].

The targeted sequencing of the coding region, intron-exon boundaries, and 3’ and 5’ untranslated regions (UTRs) of *ADAMTS13* was performed using multiplexed NGS (Human Genome Sequencing Center, Baylor College of Medicine, Houston, USA). Probes were designed by NimbleGen using genomic coordinates of our target region based on the Reference Sequence (RefSeq) collection in the UCSC Genome Browser database. Using unique barcode-sequencing tags, library pools of 8–20 samples were captured and sequenced in parallel using the Illumina HiSeq 2000 sequencing platform (Illumina, San Diego, USA). Sequence reads were processed with the Mercury analysis pipeline [23] implemented in the DNAnexus platform. The presence of all variants was validated by Sanger sequencing analysis.

For the second replication study, the same variants were searched in 4306 patients and 4887 controls with available DNA from the MEGA study, a large Dutch population-based case-control study, which included patients with a first event of DVT and pulmonary embolism [24]. The SNVs were genotyped using custom-designed TaqMan allelic discrimination assays (Applied Biosystems, Foster City, California; S1 Table) according to manufacturer’s instructions. The flow chart of the replication studies is provided (S2 Fig).

The study was approved by the Medical Ethics Committee of the Fondazione IRCCS Ca’ Granda, Hospital Maggiore and the Ethics Committee of the Leiden University Medical Center, Leiden, The Netherlands and it has been carried out in accordance with the code of ethics of the World Medical Association (Declaration of Helsinki). All patients were aware of the content of this study and gave a written informed consent.

### Statistical Analysis

Associations between SNVs and venous thrombosis were assessed by calculating ORs and 95% confidence intervals (CI), assuming an additive model of inheritance.

The post-hoc power of the replication studies, to confirm the associations found in our previously work, was calculated using G*Power version 3.0.10 software [25], based on the allele frequencies of the selected variants (p.Val154Ile, p.Asp187His and p.Arg421Cys) in the European (Non-Finnish) population reported on ExAc browser (http://exac.broadinstitute.org/; accessed September 2016), the overall replication sample size and assuming an OR of 2 and 0.05 two-tail alpha error.

## Results

### Rare *ADAMTS13* SNVs Previously Identified in DVT Patients

Biochemical and molecular data of the patients carrier for these SNVs are reported in Table 1. The molecular data of patients n. 1–8 and the plasma ADAMTS13 activity levels of patients n. 3–8 have been previously analyzed and described by Lotta *et al*. [8]. ADAMTS13 activity levels were moderately reduced for patient n. 2 (40%) and reduced for patients n. 3 (33%) and n. 8 (29%), who resulted to be carriers of p.Asp187His, p.Arg421Cys and p.Thr1249Pro, respectively. ADAMTS13 antigen levels were slightly reduced in patients n. 3 (36%) and n. 8 (31%). All other patients showed normal ADAMTS13 antigen and activity levels. The VWF:Ag values were normal for patient n. 7 (123%) and above the normal range (40–169 IU/dL) for the other patients. FVIII:C was normal for patient n. 1 (64%) and above the normal range (50–150 IU/dL) for the other. The presence of anti-ADAMTS13 autoantibodies with or without neutralizing activity was evaluated for patient n. 8 and resulted to be negative.

**Table 1:** Rare previously identified *ADAMTS13* SNVs associated with DVT.

Pt	Sex	FVIII:C (IU/dL)	VWF:Ag (IU/dL)	AD13 Antigen (%)	AD13 Activity (%)	Exon(s)	Domain(s)	Nucleotide change(s)	Amino acid Substitution
1	F	64	ND	ND	ND	5 9	MET DIS	c.[460G>A(;) 1016C>G]; [=]	p.[Val154Ile(;) Thr339Arg]; [=]
2	M	ND	295	84	40	6	MET	c.[559G>C]; [=]	p.[Asp187His]; [=]
3	M	173	246	36	33	11	TSP-1	c.[1261C>T]; [=]	p.[Arg421Cys]; [=]
4	M	201	273	177	79	16	SPA	c.[1808A>G]; [=]	p.[Tyr603Cys]; [=]
5	M	200	177	91	100	20	TSP-3	c.[2509A>G]; [=]	p.[Asp836Gly]; [=]
6	M	188	203	135	120	22	TSP-5	c.[2773A>G]; [=]	p.[Arg925Gly]; [=]
7	F	169	123	110	88	26	CUB-1	c.[3588C>A]; [=]	p.[His1196Gln]; [=]
8	M	178	283	31	29	27	CUB-1	c.[3745A>C]; [=]	p.[Thr1249Pro]; [=]
**NR**	**-**	**60–150 IU/dL**	**40–169 IU/dL**	**40–155%**	**45–138%**	**-**	**-**	**-**	**-**

Pt, patient; FVIII:C, factor VIII coagulant activity; NR, normal range; VWF:Ag, von Willebrand factor antigen; AD13, ADAMTS13 (NM_139025); F, female; M, male; ND, not determined; MET, metalloprotease domain; DIS, disintegrin-like domain; TSP-1 thrombospondin type-1 (1–8) domains; SPA, spacer domain; CUB domains.

The mutations are reported accordingly to the guidelines of the Human Genome Variation Society (http://www.hgvs.org/varnomen; accessed September 2016). [=], Wild-type allele.

### *In Silico* Evaluation of ADAMTS13 SNVs

CADD tool analyses predicted a possible deleterious effect for seven out of nine variants (p.Val154Ile, p.Asp187His, p.Thr339Arg, p.Arg421Cys, p.Tyr603Cys, p.His1196Gln and p.Thr1249Pro) as reported in Table 2. The remaining two variants (p.Asp836Gly and p.Arg925Gly) were predicted to have a neutral effect (Table 2). A further *in silico* characterization is reported in S2 Table.

**Table 2:** *In silico* evaluation of *ADAMTS13* variants.

Variant	rs number *	CADD^†^ *in silico* prediction (C-score)
p.Val154Ile	rs369026148	24.7
p.Asp187His	rs148312697	25.6
p.Thr339Arg	rs149517360	22.8
p.Arg421Cys	rs145825553	33
p.Tyr603Cys	rs867154790	24.2
p.Asp836Gly	rs868172213	0.004
p.Arg925Gly	rs782263547	4.107
p.His1196Gln	rs782230828	14.43
p.Thr1249Pro	rs867510415	12.17

*Database of Single Nucleotide Polymorphisms (dbSNP; http://www.ncbi.nlm.nih.gov/SNP/).

^†^ Combined Annotation Dependent Depletion (CADD; http://cadd.gs.washington.edu/info). A scaled C-score greater or equal 10 indicates that SNVs are predicted to be the 10% most deleterious substitutions, a score greater or equal to 20 indicates the 1% most deleterious.

### *In Vitro* Expression Studies

WT and mutant constructs were transiently transfected alone into HEK293 cells to determine a possible effect on ADAMTS13 secretion and function.

In comparison to WT, the antigen levels were reduced for SNVs p.Val154Ile (23%; 95% [CI] 18–27), p.Arg421Cys (22%; 95% [CI] 16–28), p.Tyr603Cys (27%; 95% [CI] 24–29) and were normal for the other variants (p.Asp187His, p.Thr339Arg, p.Asp836Gly, p.Arg925Gly, p.His1196Gln and p.Thr1249Pro; Table 3). ADAMTS13 activities were reduced for the following mutants: p.Val154Ile (15%; 95% [CI] 14–16), p.Asp187His (19%; 95% [CI] 17–21), p.Arg421Cys (24%; 95% [CI] 22–26) and p.Tyr603Cys (28%; 95% [CI] 26–29) and similar to WT for the other rADAMTS13. All results are reported in Table 3.

**Table 3:** *In vitro* expression studies results.

rADAMTS13	Antigen Levels (%; 95%[CI])	Activity Levels (%; 95%[CI])
WT	100	100
p.Val154Ile	23 (18–27)	15 (14–16)
p.Asp187His	58 (40–75)	19 (17–21)
p.Thr339Arg	105 (87–123)	68 (64–71)
p.Arg421Cys	22 (16–28)	24 (22–26)
p.Tyr603Cys	27 (24–29)	28 (26–29)
p.Asp836Gly	90 (86–94)	75 (70–81)
p.Arg925Gly	147 (120–174)	157 (153–161)
p.His1196Gln	85 (64–106)	83 (80–86)
p.Thr1249Pro	110 (83–137)	71 (69–74)

The amount and the activity of the mutant recombinant (r) ADAMTS13 are reported as a percentage of WT set as 100% and 95% confidence intervals (CI). The results, are the mean of at least three independent transfections.

### Western Blotting Analysis

**Fig 1:**
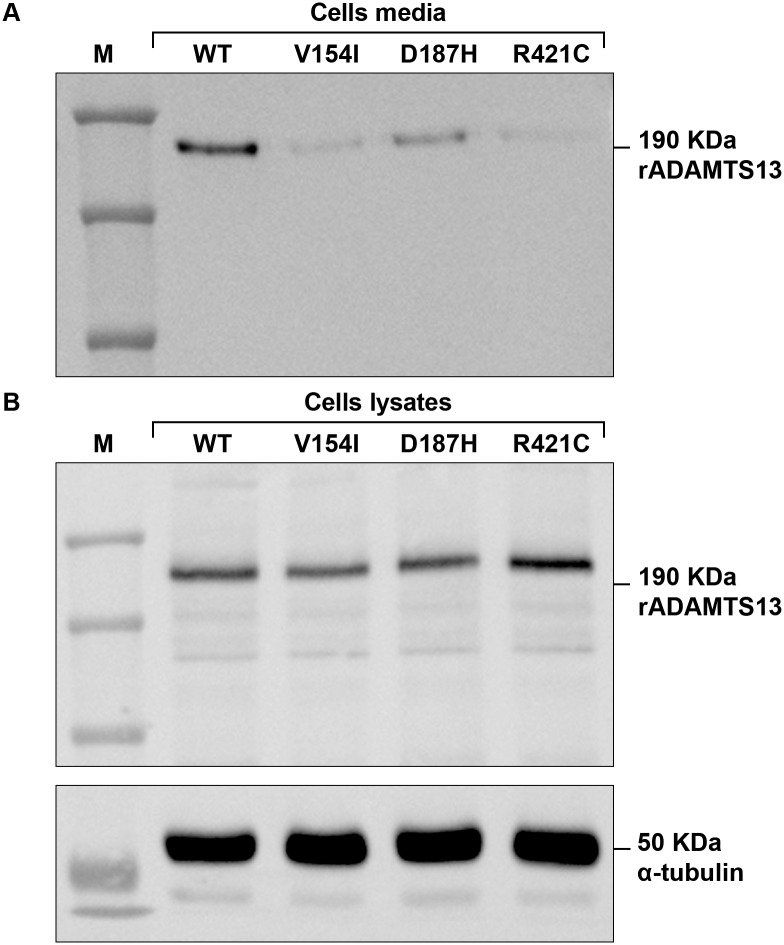
Western blot of WT and mutant p.V154I, p.D187H and p.R421C recombinant ADAMTS13 expressed in HEK293 cells. WT and mutant recombinant proteins were detected in the conditioned media (A) and cell lysates (B). Cellular alpha-tubulin was used as control to verify equal total protein loading and detected using anti-alpha-tubulin monoclonal antibody (bottom). (M) Marker. The amount of each mutant rADAMTS13 contained in cell lysates was normalized using the respective band of alpha-tubulin (loading control), quantified by densitometry analysis and referred to the WT taken as 100%.

**Fig 2:**
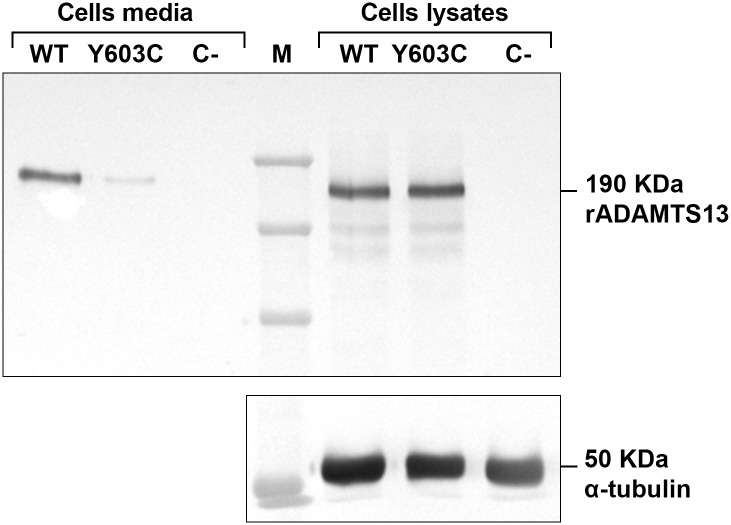
Western blot of WT and mutant p.Y603C recombinant ADAMTS13 expressed in HEK293 cells. WT and mutant recombinant protein were detected in the conditioned media (left) and cell lysates (right). Cellular alpha-tubulin was used as control to verify equal total protein loading and detected using anti-alpha-tubulin monoclonal antibody (bottom). (M) Marker; C-, medium and lysate of untransfected cells used as a negative control. The amount of each mutant rADAMTS13 contained in cell lysates was normalized using the respective band of alpha-tubulin (loading control), quantified by densitometry analysis and referred to the WT taken as 100%.

Western blot analysis was performed on conditioned media and cell lysates of transfected cells using pcDNA3.1-ADAMTS13 WT and mutant expression vectors p.Val154Ile, p.Asp187His, p.Arg421Cys and p.Tyr603Cys. In both conditioned media (Figs 1A and 2 left) and cell lysates (Figs 1B and 2 right) a band with a molecular weight of approximately 190 KDa was present. In cell lysates, also bands with lower molecular weight (~170–180 KDa) were visible. The band was not detectable in the cell media or lysate of untransfected cells used as a negative control (Fig 2).

The amount of mutant recombinant ADAMTS13 proteins in cell lysates were similar to WT with a slightly increased retention of 10–20% compared to WT for p.Val154Ile, p.Asp187His, p.Arg421Cys and a slightly reduced intracellular content (86% vs 100% of the WT) for p.Tyr603Cys. For each mutant, the detected band in the conditioned media was less intense than WT.

### Replication Studies

To validate the association of the selected *ADAMTS13* variants p.Val154Ile, p.Asp187His and p.Arg421Cys, two replication studies were carried out.

In the first replication study, performed in the Italian study, the p.Arg421Cys (c.1261C>T) allele was identified in three subjects (2/298 cases and 1/298 controls) with an [OR] of 2 (95% [CI] 0.18–22.25). Coverage and quality of the variant were similar in the two cases and one control (47X, 51X and 44X, respectively; Phred score of 60 in all three individuals). The median coverage of the target nucleotide was 48X in the sampled population (with lower limit of 14X and upper limit of 126X). The overall MAF was 0.25% (0.34% in cases vs 0.17% in controls). The presence of p.Argr421Cys was confirmed by Sanger sequencing in all subjects. The other two variants (p.Val154Ile, p.Asp187His) were not found.

In the second replication study, performed in Dutch individuals, p.Asp187His (c.559G>C) allele was found in two subjects (1/4306 case and 1/4887 control) with an [OR] of 1.14 (95%[CI] 0.07–18.15). The p.Arg421Cys allele was identified in 10 subjects (4/4306 cases and 6/4887 controls) with an [OR] of 0.76 (95%[CI] 0.21–2.68). There were no carriers of p.Val154Ile (c.460G>A). The characteristics of genotyped variants and the minor allele frequencies of each variant of both replication studies were reported in Table 4.

**Table 4:** Characteristics of genotyped single nucleotide variants (SNVs) in Italian and Dutch populations.

					Replication in Milan study			Replication in MEGA study			
Variant	rs number*	position ^†^	Major allele	Minor allele	MAF (%)	MAF Cases (%)	MAF Controls (%)	MAF (%)	MAF Cases (%)	MAF Controls (%)	ExAc Allele frequency ^‡^ (%)
p.Val154Ile	rs369026148	136291103	G	A	-	-	-	-	-	-	**0.005**
p.Asp187His	rs148312697	136291338	G	C	-	-	-	0.01	0.01	0.01	**0.064**
p.Arg421Cys	rs145825553	136298777	C	T	0.25	0.34	0.17	0.05	0.04	0.06	**0.077**

*Database of Single Nucleotide Polymorphisms (dbSNP;: http://www.ncbi.nlm.nih.gov/SNP/).

^†^ Genomic coordinates are based on build GRCh37/hg19 and refers to the position on chromosome 9.

^**‡**^ Exome Aggregation Consortium (ExAC; http://exac.broadinstitute.org/). Allele frequency reported for each variant are referred to the European (Non-Finnish) population.

Given the overall replication sample size, we calculated the post-hoc power to replicate the identified variants p.Val154Ile, p.Asp187His and p.Arg421Cys, based on the allele frequencies in the European (Non-Finnish) population reported on ExAc browser, assuming an [OR] of 2. Power to detect nominally significant evidence (*P* ≤ 0.05) was low for all variants: 8%, for p.Val154Ile, 22% for p.Asp187His and 27% for p.Arg421Cys.

## Discussion

In this study, we report the results of the *in vitro* expression of nine rare *ADAMTS13* SNVs, previously identified in eight Italian patients affected with idiopathic DVT [8]. The functional effect of these *ADAMTS13* SNVs was evaluated by performing: (i) the assessment of the SNVs potential damaging effect using CADD and (ii) the *in vitro* expression studies in HEK293 cells and patients’ plasma measurements of ADAMTS13 levels. Therefore, the three SNVs (p.Val154Ile, p.Asp187His and p.Arg421Cys), which showed a clear functional effect on ADAMTS13 levels both in patients’ plasma and *in vitro*, were selected for the replication studies in the Italian and Dutch population. The remaining SNVs were excluded due to the inconsistency between *ex vivo* and *in vitro* ADAMTS13 activity (p.Tyr603Cys and p.Thr1249Pro) or because they showed normal ADAMTS13 level both *ex vivo* and *in vitro* (p.Thr339Arg, p.Asp836Gly, p.Arg925Gly and p.His1196Gln).

*In silico* prediction performed by CADD were in agreement with our *in vitro* results for all variants, but three. Indeed, variants p.Thr339Arg, p.His1196Gln and p.Thr1249Pro, were predicted to be damaging, although *in vitro* results showed normal ADAMTS13 levels.

The *ADAMTS13* SNVs p.Val154Ile, p.Asp187His and p.Arg421Cys showed reduced *in vitro* expression levels comparable with patients’ plasma level with one missing plasma sample (patient n. 1 carrier of p.Val154Ile). Patient n. 1 was also a carrier of p.Pro618Ala, a known polymorphism associated with reduced ADAMTS13 levels [26]. However, the association of p.Pro618Ala with DVT was already evaluated and excluded [27].

The importance of Asp187 residue was previously described by *Gardner et al*. and *Pozzi et al*., who showed by *in vitro* and *in silico* experiments, respectively that the Asp187 residue stabilizes a Ca^2+^ high affinity binding site of ADAMTS13, resulting in a correct binding to VWF and proteolysis [28,29]. Recently, De Cock and colleagues reported the *in vitro* expression and characterization of p.Asp187His identified in a woman affected with congenital TTP [30]. Moreover, de Vries *et al*. described the association of p.Asp187His with the reduction of ADAMTS13 activity in adult patients affected with chronic disease [31].

The same authors, reported the association between other 4 SNVs (p.Ala140Ala, p.Arg732Val, p.Gly982Arg and p.Arg1060Trp) and the reduction of ADAMTS13 activity. Interestingly, p.Gly982Arg was identified in patients, but not in controls. However, none of these variants resulted to be associated with DVT in our Italian cohort. Two variants, p.Tyr603Cys and p.Thr1249Pro, showed inconsistencies between *in vitro* results and patients’ plasma ADAMTS13 levels. Patient n. 4 (carrier of p.Tyr603Cys) had normal plasma ADAMTS13 levels, although *in vitro* results of p.Tyr603Cys showed more than 70% reduction in ADAMTS13 levels. This could be due to the difference of *ex vivo/in vitro* secretion pathways or to an limitation of the assay which not completely reproduce the physiological conditions. Conversely, patient n. 8 (carrier of p.Thr1249Pro) showed reduced ADAMTS13 levels, whereas *in vitro* expression levels were normal. The absence of autoantibodies with or without neutralizing activity excluded that the discrepancy was due to an increased clearance of ADAMTS13 *in vivo* or to the inhibition of ADAMTS13 activity. Nevertheless, the presence of additional mutations localized in regulatory regions not covered by the NGS design should be also considered. Both patients, had additional reported polymorphisms already expressed *in vitro* by others [26,32]. Patient n. 4 was heterozygous carrier for p.Ala140Ala, Thr572Thr and p.Thr1407Thr, whereas patient n. 8 was homozygous carrier for p.Ala140Ala, p.Gln448Glu and p.Thr1407Thr. Although, Plaimauer *et al*., demonstrated that *ADAMTS13* polymorphisms might be either positive or negative modifiers of ADAMTS13 expression, a large effect these polymorphisms on patients’ phenotype is unlikely.

The latter four SNVs (p.Thr339Arg, p.Asp836Gly, p.Arg925Gly, p.His1196Gln) showed normal ADAMTS13 levels, either *in vitro* and in patients’ plasma, thus confirming their neutral effect on protein synthesis and function.

Our first study [8] had a small sample size, not allowing the individual testing of genetic variants. Therefore, we carried out two replication studies in two larger populations in order to validate the association of the selected variants p.Val154Ile, p.Asp187His and p.Arg421Cys, which showed reduced ADAMTS13 activity *ex vivo* and *in vitro*.

In the first replication study performed in Italian individuals, the p.Arg421Cys allele was identified in two patients and one control with an overall MAF of 0.25% similar to that of the original study (0.3%) [8]. Conversely, the p.Val154Ile and p.Asp187His alleles were not identified.

The second replication study, was performed in a larger Dutch population. Even in this population no carriers for the p.Val154Ile allele were found. The p.Asp187His allele was only found in 1 case and 1 control, whereas p.Arg421Cys allele was identified in 4 cases and 6 controls. Both p.Asp187His and p.Arg421Cys showed a lower overall MAF (0.01% and 0.05%, respectively) than those of the original study. These findings are in agreement with those reported by other authors, who showed how rare variants are more likely to be population specific [33]. The low allele frequencies of these variants, among Dutch individuals, is further confirmed by their absence in the Genome of the Netherlands (GONL; http://www.nlgenome.nl/; accessed September 2016), which collects the genotype data of 769 Dutch individuals.

We estimated a low post-hoc power to validate the association of these SNVs with DVT for all the three SNVs, which is a plausible explanation for the negative replication. A possible limitation of this study might be the criteria adopted for the replication studies, which are limited to specific *ADAMTS13* SNVs which cause a reduction of the ADAMTS13 activity, *ex vivo* and *in vitro*, and do not consider other coding *ADAMTS13* SNVs. In conclusion, this study confirmed a functional effect for the *ADAMTS13* SNVs p.Val154Ile, p.Asp187His, p.Arg421Cys with a moderate reduction of ADAMTS13 activity measured *in vitro* and *ex vivo*. The two replication studies confirmed a very rare allele frequency of these variants, particularly in the Dutch population, which showed lower MAF than those of the two Italian populations studied. As consequence, it remains difficult to draw a firm conclusion towards their association with DVT.

## Supporting Information

S1 Fig Flow chart of *in vitro* expression studies. (PDF)

S2 Fig Flow chart of the two replication studies. (PDF)

S3 Fig Original uncropped and unadjusted blots. (PDF)

S1 Table Taqman assay probes. (PDF)

S2 Table *In silico* tools predictions. (PDF)
