# Supplementary figures and images for: Next-Generation Sequencing and In Vitro Expression Study of ADAMTS13 Single Nucleotide Variants in Deep Vein Thrombosis
Source: PLoS One. 2016 Nov 1;11(11):e0165665. doi: 10.1371/journal.pone.0165665 (PMC5089687; doi:10.1371/journal.pone.0165665)

**Fig. S1. Flow chart of *in vitro* expression studies.**

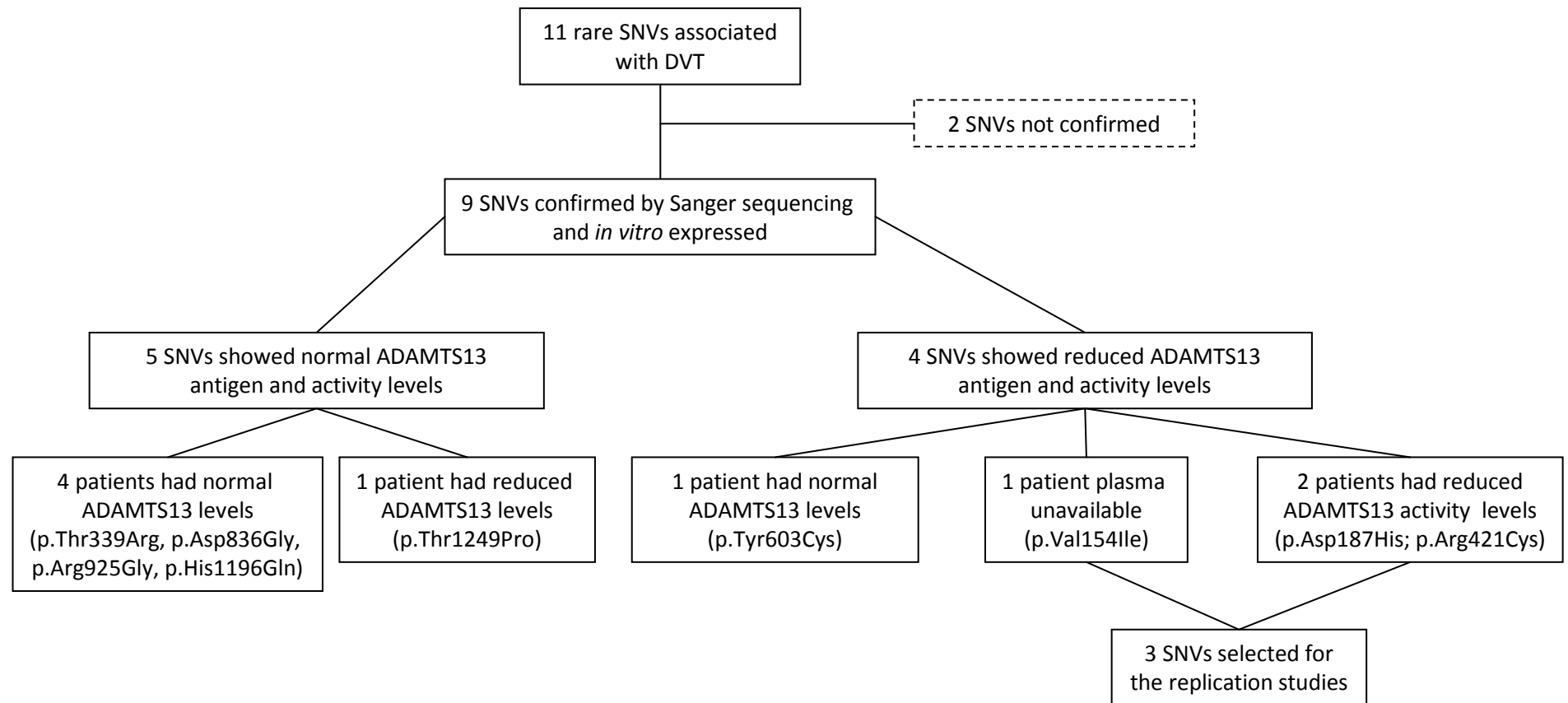

Supplement: S1 Fig — (PDF) [file pone.0165665.s001.pdf]

**Fig. S2. Flow chart of the two replication studies.**

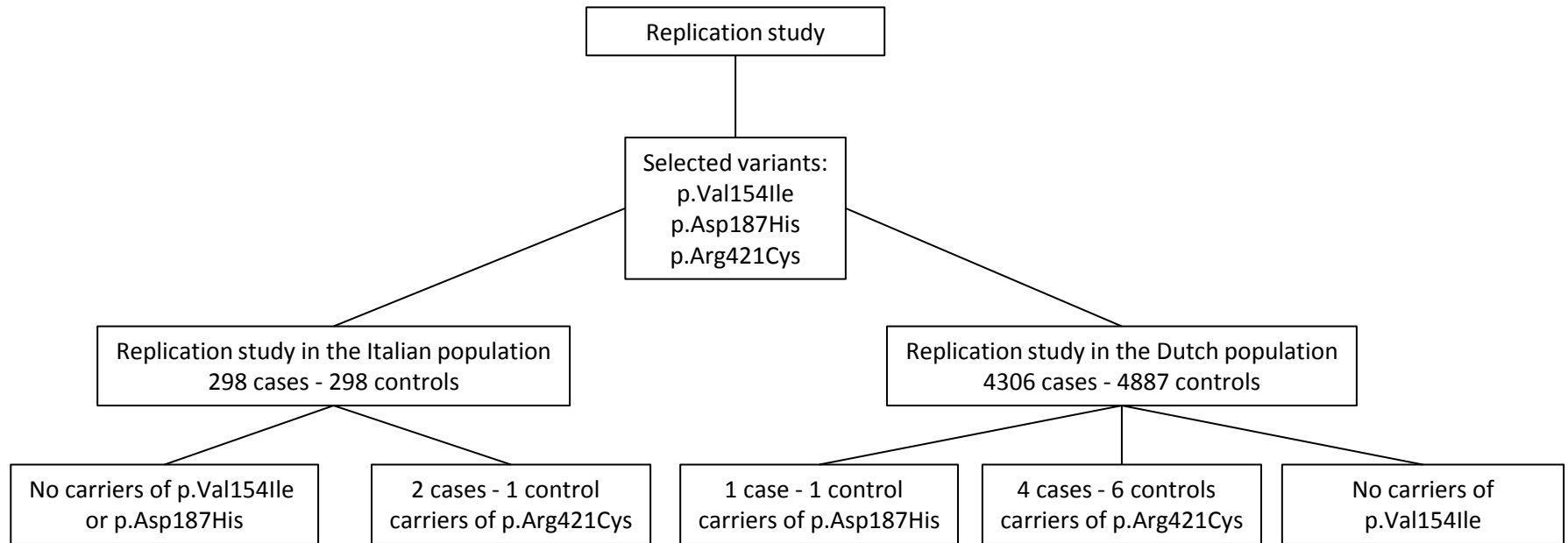

Supplement: S2 Fig — (PDF) [file pone.0165665.s002.pdf]
