# Supplementary material for: Next-Generation Sequencing and In Vitro Expression Study of ADAMTS13 Single Nucleotide Variants in Deep Vein Thrombosis
Source: PLoS One. 2016 Nov 1;11(11):e0165665. doi: 10.1371/journal.pone.0165665 (PMC5089687; doi:10.1371/journal.pone.0165665)

S3 Fig. Original uncropped and unadjusted blots.

Fig 1, panel A, uncropped blot of WT and mutant p.V154I, p.D187H and p.R421C recombinant ADAMTS13 expressed in HEK293 cells. WT and mutant recombinant proteins were detected in the conditioned media. M, marker.
Each sample has been loaded twice. The samples reported in Figure 1, panel A are those on the right.

WT V154I D187H R421C M WT V154I D187H R421C

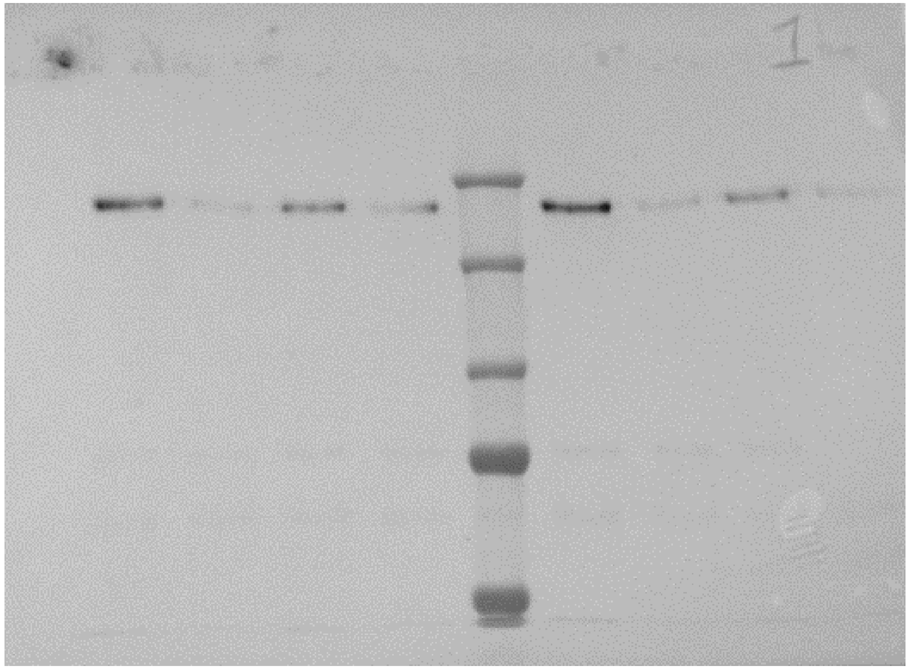

S3 Fig. Original uncropped and unadjusted blots.

Fig 1, panel B, uncropped blot of WT and mutant p.V154I, p.D187H and p.R421C recombinant ADAMTS13 expressed in HEK293 cells. WT and mutant recombinant proteins were detected in the conditioned media and cell lysates. The samples reported in Figure 1, panel B are those of the cell lysates (right). Cellular alpha-tubulin was used as control to verify equal total protein loading and detected using anti-alpha-tubulin monoclonal antibody (bottom). (M) Marker. The amount of each mutant rADAMTS13 contained in cell lysates was normalized using the respective band of alpha-tubulin (loading control), quantified by densitometry analysis and referred to the WT taken as 100%.

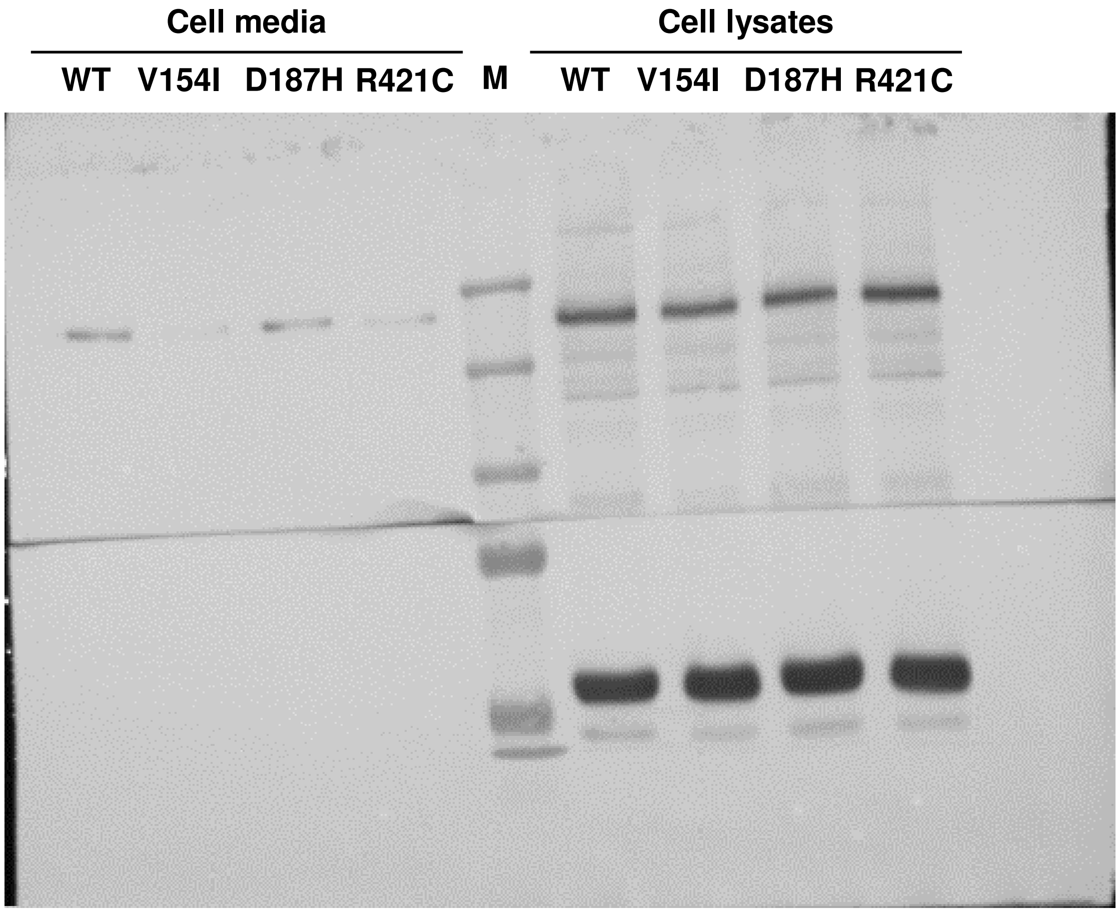

S3 Fig. Original uncropped and unadjusted blots.

Fig 2. Uncropped blot of WT and mutant p.Y603C recombinant ADAMTS13 expressed in HEK293 cells. WT and mutant recombinant protein were detected in the conditioned media (left) and cell lysates (right). Cellular alpha-tubulin was used as control to verify equal total protein loading and detected using anti-alpha-tubulin monoclonal antibody (bottom). (M) Marker; C-, medium and lysate of untransfected cells used as a negative control. The amount of each mutant rADAMTS13 contained in cell lysates was normalized using the respective band of alpha-tubulin (loading control), quantified by densitometry analysis and referred to the WT taken as 100%.

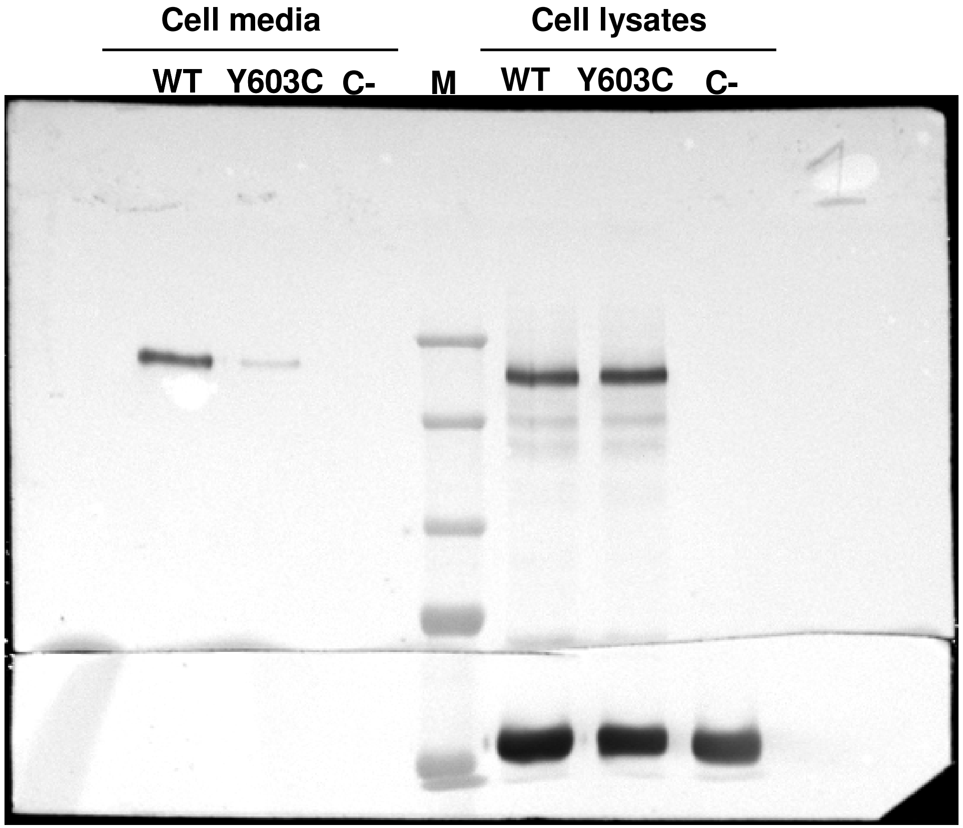

Supplement: S3 Fig — (PDF) [file pone.0165665.s003.pdf]
