# Supplementary material for: Next-Generation Sequencing and In Vitro Expression Study of ADAMTS13 Single Nucleotide Variants in Deep Vein Thrombosis
Source: PLoS One. 2016 Nov 1;11(11):e0165665. doi: 10.1371/journal.pone.0165665 (PMC5089687; doi:10.1371/journal.pone.0165665)
Supplement: S1 Table — (PDF) [file pone.0165665.s004.pdf]

Table S1. Taqman assay probes

Variant	rs number*	Forward Primer (5' to 3')	Reverse Primer (5' to 3')	VIC probe (5' to 3')	FAM probe (5' to 3')
p.Val154Ile	rs369026148	CCCCAAATATCACAGCCAACCT	TCGTCCTCAGGGTTGATGGT	ACCCACAGATGCTCAG	CCCACAGACGCTCAG
p.Asp187His	rs148312697	TGTTTTCTCTCACCGAGGTTTGAC	ACGCCCCGCACCTG	CGGTTACCATGAGGCAA	CGGTTACCATCAGGCAA
p.Arg421Cys	rs145825553	CTGTCTGCTGGGCATTTTCAG	CCTGGAGGTCAGCACCAA	ACATGCACGCCCCCCA	ACATGCACACCCCCCA

*Database of Single Nucleotide Polymorphisms (dbSNP; http://www.ncbi.nlm.nih.gov/SNP/ accessed on September 2016).
