# Supplementary material for: Next-Generation Sequencing and In Vitro Expression Study of ADAMTS13 Single Nucleotide Variants in Deep Vein Thrombosis
Source: PLoS One. 2016 Nov 1;11(11):e0165665. doi: 10.1371/journal.pone.0165665 (PMC5089687; doi:10.1371/journal.pone.0165665)
Supplement: S2 Table — (PDF) [file pone.0165665.s005.pdf]

**Table S2:** ***In silico*** **tools predictions.**

| Variant | rs number * | SIFT (<0.05 damaging effect) | PolyPhen 2.0 (Max=1) | PROVEAN (< -2.5 deleterious) | ALIGN GVGD (Max=C65) | PANTHER (>0.5 deleterious) | Consensus |
|---|---|---|---|---|---|---|---|
| p.Val154Ile | rs369026148 | 0.126 | 0.979 | -0.232 | C25 | 0.1622 | neutral |
| p.Asp187His | rs148312697 | 0.016 | 1.000 | -3.749 | C65 | n.a.† | deleterious |
| p.Thr339Arg | rs149517360 | 0.159 | 0.999 | -3.592 | C65 | 0.5089 | deleterious |
| p.Arg421Cys | rs145825553 | 0.001 | 1.000 | -5.298 | C65 | 0.80785 | deleterious |
| p.Tyr603Cys | rs867154790 | 0.000 | 1.000 | -7.777 | C65 | 0.79729 | deleterious |
| p.Asp836Gly | rs868172213 | 0.469 | 0.003 | -0.375 | C65 | 0.26159 | neutral |
| p.Arg925Gly | rs782263547 | 0.402 | 0.004 | -2.143 | C65 | 0.32161 | neutral |
| p.His1196Gln | rs782230828 | 0.211 | 0.437 | -2.108 | C15 | n.a.† | neutral |
| p.Thr1249Pro | rs867510415 | 0.012 | 0.981 | -3.086 | C35 | n.a.† | deleterious |

Summary of the single nucleotide variants (SNV) predicted as tolerated or deleterious by computational methods: SIFT (S2_File.docPolyPhen (http://genetics.bwh.harvard.edu/pph2/), PROVEAN (http://provean.jcvi.org/index.php), ALIGN GVGD (http://agvgd.hci.utah.edu/agvgd_input.php) and PANTHER (http://pantherdb.org/). All tools were used in default online mode.

*Database of Single Nucleotide Polymorphisms (dbSNP; http://www.ncbi.nlm.nih.gov/SNP/). †n.a., not applicable: the position does not align to the hidden Markov model (HMM). All tools were used in default online mode.

The consensus was obtained by considering a variant deleterious, if at least two different *in silico* tools predict a certain degree of deleteriousness.
